# Hybrid PET/MR Imaging and Brain Connectivity

**DOI:** 10.3389/fnins.2016.00064

**Published:** 2016-03-01

**Authors:** Marco Aiello, Carlo Cavaliere, Marco Salvatore

**Affiliations:** IRCCS SDN, Istituto Ricerca Diagnostica NucleareNaples, Italy

**Keywords:** PET, MR, PET/MR, brain connectivity, metabolic networks, resting state networks, connectome

## Abstract

In recent years, brain connectivity is gaining ever-increasing interest from the interdisciplinary research community. The study of brain connectivity is characterized by a multifaceted approach providing both structural and functional evidence of the relationship between cerebral regions at different scales. Although magnetic resonance (MR) is the most established imaging modality for investigating connectivity *in vivo*, the recent advent of hybrid positron emission tomography (PET)/MR scanners paved the way for more comprehensive investigation of brain organization and physiology. Due to the high sensitivity and biochemical specificity of radiotracers, combining MR with PET imaging may enrich our ability to investigate connectivity by introducing the concept of metabolic connectivity and cometomics and promoting new insights on the physiological and molecular bases underlying high-level neural organization. This review aims to describe and summarize the main methods of analysis of brain connectivity employed in MR imaging and nuclear medicine. Moreover, it will discuss practical aspects and state-of-the-art techniques for exploiting hybrid PET/MR imaging to investigate the relationship of physiological processes and brain connectivity.

## Introduction

Currently, one of the main challenges in neuroscience is to gain an understanding of how brain activity generates behavior and, in more detail, how neural information is segregated and integrated. Anatomical and physiological studies support the idea that cognitive processes depend on interactions among distributed neuronal populations and brain regions (Sporns, 2013); brain connectivity (BC) refers to the structural and functional characterization of those interactions. Four different but related types of connectivity are currently being investigated by neuroscientists: anatomical or structural, functional, metabolic and effective connectivity (Horwitz, 2003; Lee et al., 2003).

Neuroimaging plays a crucial role in BC investigation and magnetic resonance (MR) imaging (MRI) is commonly used to assay structural and functional connectivity, comprising the majority of *in vivo* BC studies (Buckner et al., 2013). The recent development of hybrid positron emission tomography (PET)/MR scanners allows for more thorough investigation of BC and its underlying physiological processes (Wehrl et al., 2013; Riedl et al., 2014; Aiello et al., 2015; Tahmasian et al., 2015).

PET integrated with MRI yields both high sensitivity and specificity due to the use of radiotracers. [18F] fluorodeoxyglucose (FDG) is the most widely used radiotracer, primarily for estimating glucose consumption in both neurological and oncological studies. Previous studies have demonstrated a close linkage between functional activity, blood flow, and glucose utilization (Jueptner and Weiller, 1995). Glucose metabolism, as estimated by FDG-PET, and oxygen consumption, as estimated by functional MRI (fMRI), are in turn related to cerebral blood flow (CBF), which delivers O_2_ and glucose to the brain (Attwell and Iadecola, 2002).

The across-subjects covariance of FDG-PET images yields a measure of metabolic connectivity that relates to functional connectivity due to the physiological coupling of glucose and oxygen metabolism. This has led to an increase in the importance of PET/MRI as a multi-modality probe of BC, resulting in the development of the concept of cometomics, which combines connectivity with metabolic information (Wehrl et al., 2014).

This review will provide the reader with a comprehensive taxonomy of different BC methods involved in PET/MRI and the techniques used to employ those methods.

## Brain connectivity

Brain Connectivity involves anatomical pathways and interactions, as well as communication between distinct units of the central nervous system. These units are categorized into micro-(individual neurons), meso-(columns), or macro-(regions) scales (Pawela and Biswal, 2011). Notably, the imaging methods presented here provide suitable non-invasive estimations of different aspects of BC, but “actual” BC is generally determined only through the use of invasive techniques or histological assessment of *ex vivo* or post-mortem samples (Thomas et al., 2014).

Before the recent explosion of BC studies, a great deal of brain mapping consisted of functional segregation and localization of those functions; the current trend reflects a paradigm shift from segregation to integration wherein functional segregation must be viewed in the context of functional integration and *vice versa* (Friston, 2011). The theory of functional integration rests on the analysis of how different regions in a neuronal system interact, and results in the identification of networks of interactions among specialized brain areas. Once identified, such networks can be also characterized by analysis of their topological properties (Fornito et al., 2013; Sporns, 2014).

BC is a multifaceted concept: it can be subdivided into structural and functional domains, each further divided into static and dynamic components (Pawela and Biswal, 2011; Lang et al., 2012). Static connectivity can be measured by anatomical properties using a number of imaging methods, including high-resolution MRI (Mechelli et al., 2005; Alexander-Bloch et al., 2013), PET imaging, diffusion tensor imaging (DTI), and histology (myelination). Dynamic connectivity can be measured by a wide variety of techniques, particularly those with a fast time scale capable of measuring causality, such as EEG and MEG, or that can provide information about the spatial distribution and strength of connections, such as resting-state functional connectivity MRI (rs-fcMRI).

The technological development of imaging modalities, especially with the advent of hybrid scanners, along with the advancement of computation methods aimed to infer different aspects of BC, are boosting BC research. The proliferation of multiple methods for the estimation of BC requires the user to be aware of the specific details of the method and its appropriate neurophysiological interpretation. For example, in the case of functional connectivity (FC), deriving the same conclusions from multiple techniques it is not trivial. It is often unclear which aspects of co-varying neural activity are being assessed by any particular computation of FC (Horwitz, 2003). The following sections will provide a taxonomy of BC methods involved in PET/MR studies: structural connectivity via diffusion tractography (DT), FC via resting state functional MRI (rs-fMRI), and metabolic connectivity via FDG-PET, with a particular emphasis on metabolic connectivity, as it is strictly related to PET imaging and therefore less commonly reviewed than structural and FC.

### Structural connectivity

Structural Connectivity (SC) is defined as the map of structural cortical/subcortical connections (connectome) and is derived mainly from diffusion-weighted imaging (DWI). Although this concept is broadly accepted, many issues regarding acquisition, processing, visualization and integration of SC with other BC maps remain debated.

Many acquisition parameters can affect data quality, including the number of directions investigated and the maximum b-value used (Mori and Zhang, 2006). Moreover, issues linked to specific patients populations (e.g., brain anatomy distortion, cerebral edema, or white matter lesions) have to be taken into account in the determination of SC as for other measures of BC (Cavaliere et al., 2014). Image processing to create SC maps can involve employing different tractography algorithms and diffusion models, including a deterministic or probabilistic approach, locally greedy or globally optimal processing, and single- or multi-diffusion model. The choice of diffusion model affects network properties of SC maps (Bastiani et al., 2012). After fiber reconstruction, the number of trajectories or other voxel-wise indices of fiber integrity, such as fractional anisotropy or mean diffusivity, can be used to weight the connectivity strength between brain regions. Other, more complex, measures to quantify connection weights include estimation of axon diameter and density using tailored DWI acquisitions (Alexander et al., 2010) or analysis of myelination by magnetization transfer imaging (van den Heuvel et al., 2009). The covariance across subjects of morphological properties (such as atrophy and cortical thickness) between brain regions is also relevant to SC; this is usually referred as structural covariance (Mechelli et al., 2005; Lerch et al., 2006; Alexander-Bloch et al., 2013) and has been demonstrated to be suitable for understanding various pathological conditions (Bernhardt et al., 2014; Valk et al., 2015). Methodologically speaking, structural covariance is estimated using high-resolution MRI and, given the common static feature, can be achieved with the same approaches employed in investigating metabolic connectivity as described in Section Metabolic Connectivity.

### Functional connectivity

FC is defined as “temporal correlations between spatially remote neurophysiological events” (Friston et al., 1993a) and was first described using multiunit systems of neural activity recording and subsequently by tomographic imaging with ^15^O-PET activation studies (Friston et al., 1993b). The wide, interdisciplinary increase in FC studies in neuroimaging began after the identification of two phenomena: the blood oxygenation level dependent (BOLD) signal (Ogawa et al., 1990), as measured by fMRI, which has better spatial and temporal resolution than PET imaging, and resting-state fMRI, a significant temporal correlation in fMRI signal in subjects who were not cognitively engaged (Biswal, 2012).

Raichle et al. (2001) demonstrated experimentally that the majority of the brain's energy consumption occurs at resting-state and activity increases by < 5% during focused mental tasks. This study also coined the term default mode network (DMN) to describe the most prominent resting state network (RSN) connecting the medial temporal lobe, the medial prefrontal cortex, the posterior cingulate cortex, the ventral precuneus, and parts of the parietal cortex.

FC methods possess a common statistical nature, nevertheless, each different measure may be assessing a different aspect of interregional interactions. As a result, the concept of functional and effective connectivity are extensively debated and, at times, believed “elusive” (Horwitz, 2003; Lee et al., 2003; Fingelkurts et al., 2005).

Different computational methods have been employed to investigate and define the functional relationship between distinct cortical regions. The classical and most intuitive method is seed based analysis (SBA), which consists of selecting of a region of interest (ROI) as a seed and generating a connectivity map by computing which regions or voxels are functionally connected to the seed according to a predefined metric, most commonly the Pearson correlation coefficient between BOLD time courses. SBA plays an essential role as a technique for FC estimation, as well as for independent component analysis (ICA; Himberg et al., 2004; Esposito et al., 2005). ICA evaluates the fMRI signal as a linear mixture of various signals, which predominantly originate from fluctuations of neuronal, cardiac and respiratory sources. Both SBA and ICA are successfully employed in clinical and behavioral research (Lee et al., 2013).

Other rs-fMRI-derived FC metrics that capture different FC properties in voxel-wise maps have recently been introduced. These metrics are of particular interest in PET/MRI probes (Aiello et al., 2015). The Degree of Centrality (DC; Buckner et al., 2009; Martuzzi et al., 2011; Tomasi and Volkow, 2012), employs Pearson's correlation coefficient as a metric for estimation of functional connectivity between each pair of voxels, assigning to each voxel a global number of functional connections between it and all other voxels across the brain. Regional Homogeneity (ReHo) is a voxel-based measure of brain activity that estimates the degree of synchronization between the time series of a given voxel and its nearest neighbors (Zang et al., 2004). Fractional Amplitude of Low Frequency Fluctuations (fALFF, Zou et al., 2008; Zuo et al., 2010) is equal to the power within the low-frequency range (0.01–0.1 Hz) divided by the total power in the entire detectable frequency range for the time course of each voxel and quantifies the amplitude of low frequency oscillations (LFOs), thus representing the relative contribution of LFOs to the whole frequency range. A recent PET/MR study (Tomasi et al., 2013) demonstrated that the amplitude of the fluctuations of RSNs is relevant in modeling energy consumption in FC.

The spatial extent of the physiological phenomena probed by these measures also differs. fALFF contrast is measured by single voxel signal, and is thus independent of the spatial range of the underlying connectivity processes. On the other hand, ReHo serves as a measurement of short-range functional connectivity (amongst neighboring cells) while DC is essentially weighted by long-range functional connectivity due to the fact that distant voxels far outnumber neighboring ones.

### Metabolic connectivity

Metabolic images, such as FDG-PET scans, are traditionally studied following intensity-based analysis. Metabolic connectivity (MC) aims, in general, to analyze FDG-PET data in terms of covariance across subjects.

Due to the limited temporal resolution of PET images and the steady-state nature of typical FDG-PET scans, the source of signal variability mainly lies in within-group variance, as opposed to rs-fMRI functional connectivity, where dynamical signal variations enable the study of the covariance of subjective signal fluctuations. Therefore, two regions of the brain are considered metabolically connected based on whether the estimation of their glucose consumption significantly correlates across subjects in a specific group.

As with FC, MC can be estimated using different approaches. First, Horwitz et al. (1984) proposed that anatomical regions with correlated glucose uptake values are functionally associated, and the strength of the association is proportional to the magnitude of correlation. This method, thereafter referred to as Interregional Correlation Analysis (IRCA), is principally similar to SBA and estimates the correlation between mean values of glucose metabolic rate (GMR) of pre-defined brain regions. This approach demonstrated MC networks for the first time and documented their potential as biomarkers in Alzheimer's disease and many subsequent studies confirmed its suitability for MC research (Table 1).

**Table 1:** **Summary of different approaches for the estimation of metabolic connectivity**.

**References**	**Method**	**Subjects' characteristics**	**Toolbox**
Horwitz et al., 1984, 1995; Laureys et al., 1999; Vogt et al., 2006; Lee et al., 2008; Morbelli et al., 2010, 2013a,b; Sanabria-Diaz et al., 2013; Carbonell et al., 2014; Arthuis et al., 2015	Interregional Correlation Analysis (IRCA)	Healthy subjects, Alzheimer Disease, MCI epilepsy	n/a
Huang et al., 2010; Zou et al., 2015	Sparse Inverse Covariance Estimation (SICE)	Alzheimer Disease, MCI, Human Controls	GraphVar
Di et al., 2012; Toussaint et al., 2012; Wehrl et al., 2013; Yakushev et al., 2013; Pagani et al., 2016	Spatial Independent Component Analysis (sICA)	Alzheimer Disease, MCI, Amyotrophic Lateral Sclerosis, Human Controls, Rats	Gift toolbox, NetBrainWork, Melodic
Moeller et al., 1987; Rottenberg et al., 1987; Alexander and Moeller, 1994; Eidelberg et al., 1995, 1997; Feigin et al., 2001; Kaasinen et al., 2006^*^; Nobili et al., 2008^*^; Meles et al., 2015; Spetsieris et al., 2015; Tripathi et al., 2016	Scaled Subprofile Model Principal Component Analysis (SSM-PCA)	Neuropsychiatric Disorders, Huntington's disease, Human Controls, Parkinson Disease, Dementia	ScAnVp, gCVA
Passow et al., 2015	Seed based analysis on dynamic PET data	Healthy subjects	SPM8

* *These works are based on classical principal component analysis*.

As for SBA, pairwise correlation captures a limited aspect of information and does not provide a complete account of multi-regional interactions, resulting in the development of other computational approaches for MC.

Sparse inverse covariance estimation (SICE; Huang et al., 2010; Zou et al., 2015) yields the correlation between a pair of ROIs, given all other regions, and further demonstrated the relevance of MC in AD studies.

Another global approach is the scaled subprofile model principal component analysis (SSM-PCA; Moeller et al., 1987) that was first successfully employed in AIDS dementia complex and then widely employed for MC characterization of various diseases (Table 1).

Pre-processing of FDG-PET data in terms of spatial and intensity normalization is a common denominator of the MC methods listed in Table 1. Intensity normalization (i.e., scaling of tracer uptake to a reference region) is in most cases essential for analyses of non-quantitative data, as is the case for static FDG-PET. A reference region can be chosen either pathologically, as a region not affected by brain pathology, or with data-driven approaches (Dukart et al., 2013). Alternately, z-value transformation can be employed for intensity normalization in studies of the relationship between FDG-PET and other variables (Chételat et al., 2008).

Since MC analyzes the covariance across subjects while FC analyzes covariance across timepoints, computational tools and FC methods can be used on PET data by simulating a temporal sequence of PET data from a group of subjects by concatenating spatially normalized PET scans from different subjects (Di et al., 2012). The most current approaches include temporal information from FDG-PET dynamical acquisition as well (Wehrl et al., 2013; Passow et al., 2015); in these methods covariance between glucose uptake timecourses of different regions also contributes to MC estimation.

Since the FDG-PET signal is mainly derived from gray matter, partial volume effect may confound the estimation of actual glucose uptake and, subsequently, MC. PET/MRI can exploit the anatomical detail of MRI for partial volume correction of FDG-PET data (Quarantelli et al., 2004) or gray matter density can be removed as a confounding variable by regression in a correlation analysis (Aiello et al., 2015). Although MC methods have been extensively employed with FDG-PET imaging, they are also applicable to other PET tracers (Kaasinen et al., 2006), static images such as anatomical maps from high resolution MRI (as described above for structural covariance networks) and, in principle, to metabolic maps from MR spectroscopic imaging. Though MC methods are inherently designed for group studies, some statistical approaches that compute a subject score for the corresponding disease-related pattern of MC (Niethammer and Eidelberg, 2012; Toussaint et al., 2012) revealed the potential to also employ MC in differential diagnosis.

## Neurophysiological considerations

The physiological mechanisms underlying neural activity, and thus BC, have been a matter of debate in the last century. The early principle that changes in blood flow are a function of tight coupling between energy requirements and the supplies of glucose and oxygen (Roy and Sherrington, 1890) has been contradicted by evidence from PET and fMRI experiments (Lin et al., 2010). PET imaging showed that levels of aerobic glycolysis are not strictly related to levels of brain energy metabolism, and are different across brain regions (Vaishnavi et al., 2010).

Buckner et al. (2008) also consider PET/MR imaging to provide valuable information for understanding the physiology underlying BC; since the DMN emerges from hemodynamic measures of blood flow that are indirectly linked to neural activity, they address whether or not vascular characteristics can account for the default network's anatomy. FDG-PET studies provide evidence that regions within the default network show disproportionately high resting glucose metabolism relative to other brain regions and that DMN anatomy does not rely on vascular coupling (Gusnard and Raichle, 2001; Vogt et al., 2006). These studies suggest the great potential of integrated PET/MRI studies for the investigation of the relationship between FC and glucose metabolism.

Recent studies have investigated the relationship between energy consumption and FC using *in vivo* imaging techniques, with PET and MR acquired separately (Nishida et al., 2008; Li et al., 2012; Liang et al., 2013; Tomasi et al., 2013; Passow et al., 2015; Soddu et al., 2015). Preclinical studies using a hybrid scanner (Wehrl et al., 2013) showed that FDG-PET and BOLD fMRI resting-state networks agree to some extent, but show a significantly different pattern of cortical activation. In humans, Riedl et al. (2014) demonstrated a relationship between GMR and FC changes in regions activated by visual stimuli.

**Figure 1:**
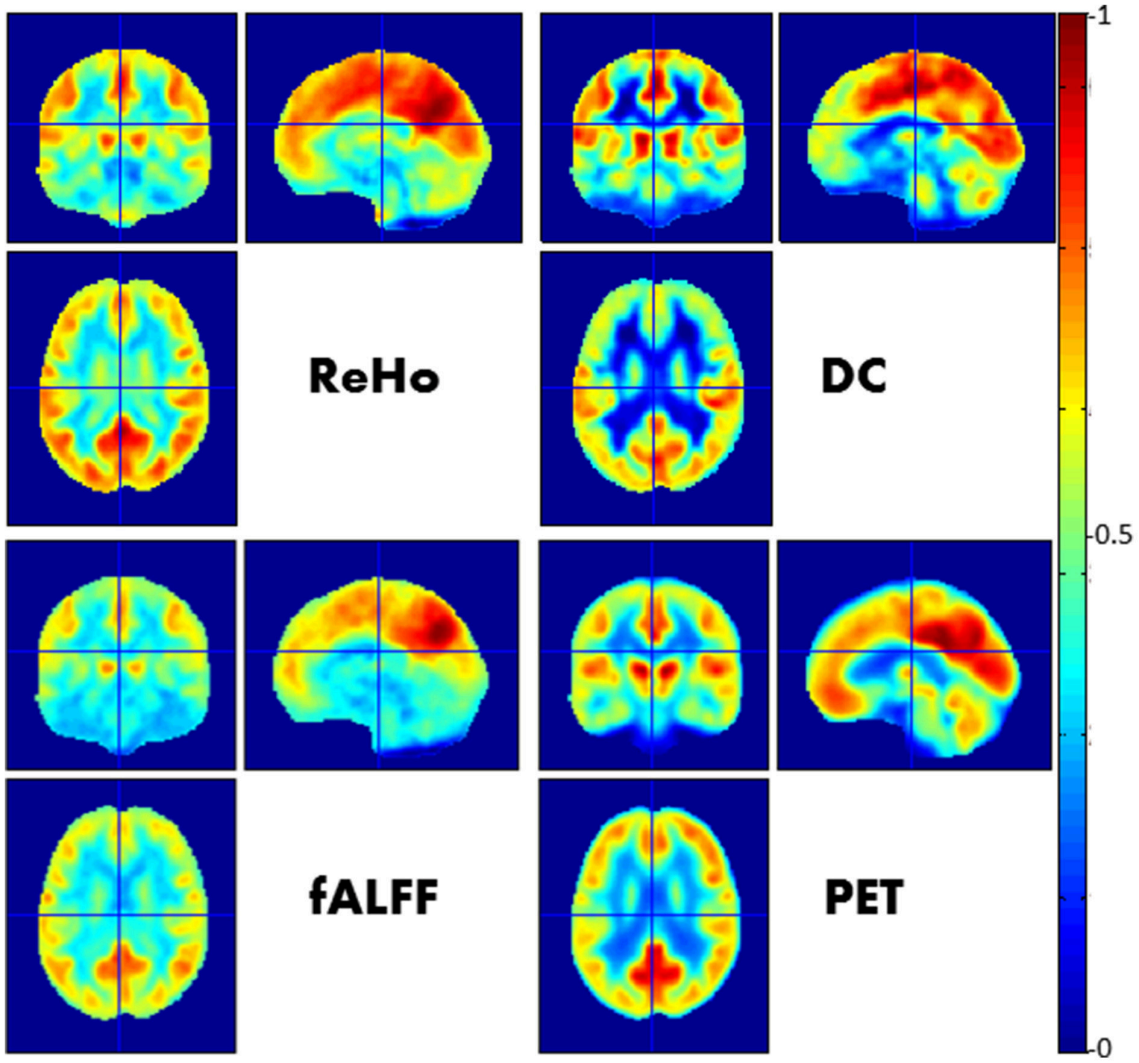
**Relationship between FC imaged by rs-fMRI and glucose metabolism imaged by FDG-PET**. In this figure a visual comparison between different voxel-wise maps of FC (namely ReHo, DC, fALFF) and PET images is presented. Each map was obtained by averaging spatially normalized maps over 23 neurologically healthy subjects and afterwards normalized with respect the maximum value. See Aiello et al. (2015) for further details.

In a simultaneous PET/MR study by Aiello et al. (2015), the authors found a heterogeneous correlation between the spatial distributions of PET and rs-fMRI-derived metrics across both anatomic regions and functional networks, with the strongest correlation for the DMN (Figure 1).

SC plays a fundamental role in higher levels of brain organization, although its underlying physiological phenomenon (e.g., water diffusion along axonal path) is certainly different than glucose and oxygen metabolism. Effectively, SC constitutes the routes of communication for FC and MC and contributes to the energetic cost of connectivity through wiring efficiency (Bullmore and Sporns, 2012; Tomasi et al., 2013; Aiello et al., 2015).

## Relationship between BC measures

The study of the relationship between different BC measures is of great interest in the neuroscience community. Rather than study each single modality in isolation, the investigation of mutual connections between BC modalities can provide valued insights into the brain's mechanisms of organization. Neuroimaging findings currently provide strong supporting for the existence of a relationship between the anatomical architecture of the brain and RSNs (Honey et al., 2009; Das et al., 2014; Goñi et al., 2014; Wang et al., 2014). Neuroimaging studies have shown a close linkage between SC and FC in other brain systems besides the DMN, such as the executive control network, salience network and primary motor and visual network (van den Heuvel et al., 2009). In other cases, differences between SC and FC emerged (Wang et al., 2014); areas can show FC without a direct anatomical connection (Deco et al., 2013). Moreover, there is also scientific evidence that dynamic variations of FC occur in the human brain in fixed anatomical pathways (Hutchison et al., 2013). These findings suggest that the FC-SC relationship needs to be modeled as a complex, dynamic system (Deco et al., 2013; Das et al., 2014; Minati et al., 2015).

According to the economic principle of the brain, minimizing wiring and metabolic energy costs results in a more efficient tradeoff between wiring costs and extent of structural and/or functional connectivity among spatially distinct brain regions (Das et al., 2014). However, there is strong evidence that metabolic costs are controlled dynamically within an upper limit imposed by the anatomical architecture of the network. Brain networks are often functionally activated or configured less expensively than they could be within anatomical constraints, to ensure the frugal use of metabolic resources (Bullmore and Sporns, 2012). Diaschisis further supports the idea of MC-SC coupling (Feeney and Baron, 1986; Sestini et al., 2010).

## Practical considerations

Simultaneous PET/MR imaging allows acquisition of different biological properties (e.g., glucose/oxygen metabolism or perfusion/glucose metabolism) at the same time, and therefore likely under the same physiological conditions. This alleviates problems associated with capturing multiple parameters on different time scales. While FDG-PET measures glucose uptake integrated over minutes, starting from radiotracer administration, FC is estimated by rs-fMRI acquired each few seconds. There are different approaches to managing this discrepancy, both with simultaneous and sequential scanning: off-scanner injection of radiotracer (Tomasi et al., 2013; Aiello et al., 2015; Passow et al., 2015), following clinical recommendations for neurological FDG-PET imaging, or in-MR injection, where FDG is administered during MRI acquisition (Newberg et al., 2005; Musiek et al., 2012; Chonde et al., 2013).

Regarding the spatial correspondence between PET and MR, the inherent co-registration carried out by simultaneous PET/MR imaging is particularly useful when the pattern of a specific PET tracer (such as [18F]choline, [11C]raclopride, or [18F]DOPA) does not completely reflect the brain anatomy, causing potential failure of retrospective fusion algorithms.

Another advantage of simultaneous PET/MRI in BC studies lies in the ability to mitigate the problem of blurred PET images due to the subjects' motion, which is often a dramatic problem in cases of dementia, movement disorders and disorders of consciousness (Soddu et al., 2011). With simultaneous PET/MRI, PET data can be motion corrected by exploiting the high temporal resolution of simultaneously acquired rs-fMRI (Catana et al., 2011).

Current MR-based attenuation correction (AC) produces slightly spatially biased metabolic patterns relative to CT-based AC (Andersen et al., 2014; Hitz et al., 2014), with significantly lower PET values in fronto-parietal portions of the neocortex, and significantly higher values in subcortical and basal regions of the brain. These differences must be taken into account during the interpretation of glucose metabolism distribution from PET/MRI studies.

## Conclusions

The concept of the brain connectivity has been reviewed in light of opportunities presented by PET/MRI. The aim was to provide to the reader with an overview of methodological issues arising from different fields of MRI and nuclear medicine. From critical analysis of the scientific literature, one can see the importance of a leading role in neuroimaging studies for PET/MRI.

## Author contributions

MA: Conceived the work, wrote and revised the manuscript. CC: Wrote and revised the manuscript. MS: Wrote and revised the manuscript.

### Conflict of interest statement

The authors declare that the research was conducted in the absence of any commercial or financial relationships that could be construed as a potential conflict of interest.
